# Diphenyl (*o*-tolyl­amido)­phospho­nate

**DOI:** 10.1107/S1600536811013924

**Published:** 2011-04-16

**Authors:** Fahimeh Sabbaghi, Mehrdad Pourayoubi, Poorya Zargaran

**Affiliations:** a Department of Chemistry, Zanjan Branch, Islamic Azad University, PO Box 49195-467, Zanjan, Iran; b Department of Chemistry, Ferdowsi University of Mashhad, Mashhad, 91779, Iran

## Abstract

The asymmetric unit of the title compound, C_19_H_18_NO_3_P, contains two independent mol­ecules in which the P atoms are found in slightly distorted tetrahedral environments. In the crystal, pairs of inter­molecular N—H⋯O(P) hydrogen bonds form two independent centrosymmetric dimers.

## Related literature

For a related structure, see: Pourayoubi *et al.* (2010 ▶).
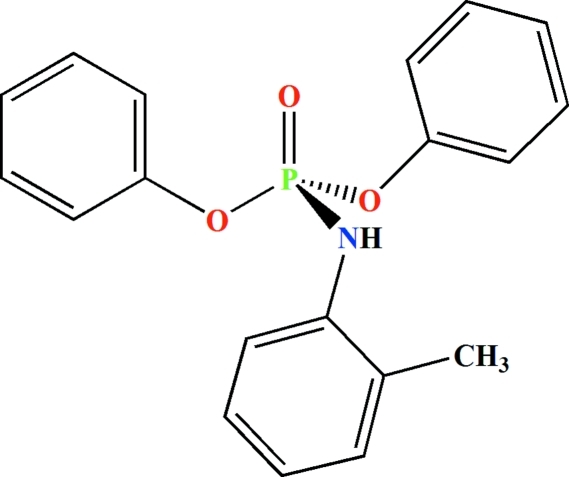


## Experimental

### 

#### Crystal data

C_19_H_18_NO_3_P
*M* *_r_* = 339.31
Triclinic, 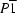

*a* = 10.2986 (13) Å
*b* = 10.3540 (12) Å
*c* = 17.993 (2) Å
α = 86.650 (2)°
β = 84.036 (2)°
γ = 62.429 (2)°
*V* = 1691.4 (4) Å^3^
*Z* = 4
Mo *K*α radiation
μ = 0.18 mm^−1^
*T* = 120 K
0.50 × 0.40 × 0.25 mm

#### Data collection

Bruker SMART 1000 CCD area-detector diffractometer
Absorption correction: multi-scan (*SADABS*; Sheldrick, 1998 ▶) *T*_min_ = 0.918, *T*_max_ = 0.956
17593 measured reflections
8135 independent reflections
6772 reflections with *I* > 2σ(*I*)
*R*_int_ = 0.018

#### Refinement

*R*[*F* ^2^ > 2σ(*F* ^2^)] = 0.041
*wR*(*F* ^2^) = 0.104
*S* = 1.00
8135 reflections
441 parameters
H atoms treated by a mixture of independent and constrained refinement
Δρ_max_ = 0.31 e Å^−3^
Δρ_min_ = −0.34 e Å^−3^

### 

Data collection: *SMART* (Bruker, 1998 ▶); cell refinement: *SAINT-Plus* (Bruker, 1998 ▶); data reduction: *SAINT-Plus*; program(s) used to solve structure: *SHELXTL* (Sheldrick, 2008 ▶); program(s) used to refine structure: *SHELXTL*; molecular graphics: *Mercury* (Macrae *et al.*, 2008 ▶); software used to prepare material for publication: *SHELXTL* and *enCIFer* (Allen *et al.*, 2004 ▶).

## Supplementary Material

Crystal structure: contains datablocks I, global. DOI: 10.1107/S1600536811013924/lh5229sup1.cif

Structure factors: contains datablocks I. DOI: 10.1107/S1600536811013924/lh5229Isup2.hkl

Additional supplementary materials:  crystallographic information; 3D view; checkCIF report

## Figures and Tables

**Table 1:** Hydrogen-bond geometry (Å, °)

*D*—H⋯*A*	*D*—H	H⋯*A*	*D*⋯*A*	*D*—H⋯*A*
N1—H1*N*⋯O1^i^	0.85 (2)	2.09 (2)	2.903 (1)	162 (1)
N1′—H1′*N*⋯O1′^ii^	0.86 (2)	2.02 (2)	2.867 (1)	167 (1)

Symmetry codes: (i) 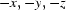; (ii) 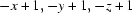.

**Fig. 1.**
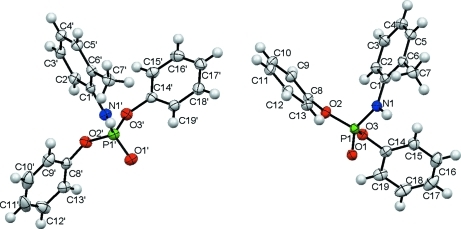
The asymmetric unit of title compound with ellipsoids drawn at the 50% probability level.

**Fig. 2.**
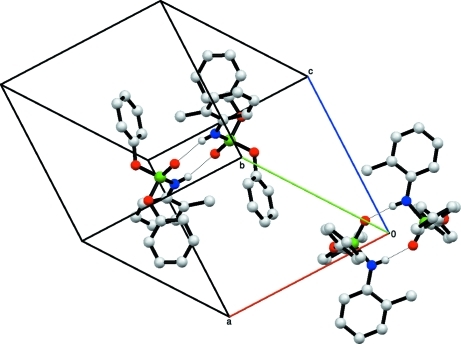
Part of the crystal structure with hydrogen bonds shown as dotted lines. Only H atoms involved in hydrogen bonds are shown.

## supplementary crystallographic information

### Comment

In previous work, the structure of diphenyl (*p*-tolylamido)phosphate was reported (Pourayoubi *et al.*, 2010). Here, we report on the synthesis and crystal structure of title compound.

The asymmetric unit (Fig. 1) consists of two independent molecules. The P═O, P—O and P—N bond lengths are standard for amidophosphoric acid ester compounds (Pourayoubi *et al.*, 2010). The P atoms of two independent molecules are in slightly distorted tetrahedral environments.

In each molecule, the phosphoryl group and the N-H unit are in a *syn* orientation with respect to each other and in the crystal, pairs of intermolecular N—H···O(P) hydrogen bonds (Table 1) form two independent centrosymmetric dimers (Fig. 2).

### Experimental

To a solution of (C_6_H_5_O)_2_P(O)Cl in chloroform, a solution of *ortho*-toluidine (1:2 mole ratio) in chloroform was added at 273 K. After 4 h stirring, the solvent was removed and product was washed with distilled water. Colorless plates were obtained by slow evaporation of a methanol solution of the title compound at room temperature.

### Refinement

The hydrogen atoms of N-H groups were found in difference Fourier maps and were refined with isotropic displacement parameters. The H(C) atom positions were calculated and refined in a riding-model approximation with *U*_iso_(H) equal to 1.2*U*_eq_(C), or 1.5 *U*_eq_(C) for methyl group H atoms.

### Crystal data

**Table:**

C_19_H_18_NO_3_P	*Z* = 4
*M**_r_* = 339.31	*F*(000) = 712
Triclinic, *P*1	*D*_x_ = 1.332 Mg m^−^^3^
Hall symbol: -P 1	Mo *K*α radiation, λ = 0.71073 Å
*a* = 10.2986 (13) Å	Cell parameters from 512 reflections
*b* = 10.3540 (12) Å	θ = 4.5–56.0°
*c* = 17.993 (2) Å	µ = 0.18 mm^−^^1^
α = 86.650 (2)°	*T* = 120 K
β = 84.036 (2)°	Plate, colorless
γ = 62.429 (2)°	0.50 × 0.40 × 0.25 mm
*V* = 1691.4 (4) Å^3^	

### Data collection

**Table:**

Bruker SMART 1000 CCD area-detector diffractometer	8135 independent reflections
Radiation source: fine-focus sealed tube	6772 reflections with *I* > 2σ(*I*)
graphite	*R*_int_ = 0.018
φ and ω scans	θ_max_ = 28.0°, θ_min_ = 2.2°
Absorption correction: multi-scan (*SADABS*; Sheldrick, 1998)	*h* = −13→13
*T*_min_ = 0.918, *T*_max_ = 0.956	*k* = −13→13
17593 measured reflections	*l* = −23→23

### Refinement

**Table:**

Refinement on *F*^2^	Primary atom site location: structure-invariant direct methods
Least-squares matrix: full	Secondary atom site location: difference Fourier map
*R*[*F*^2^ > 2σ(*F*^2^)] = 0.041	Hydrogen site location: mixed
*wR*(*F*^2^) = 0.104	H atoms treated by a mixture of independent and constrained refinement
*S* = 1.00	*w* = 1/[σ^2^(*F*_o_^2^) + (0.0458*P*)^2^ + 0.9439*P*] where *P* = (*F*_o_^2^ + 2*F*_c_^2^)/3
8135 reflections	(Δ/σ)_max_ = 0.001
441 parameters	Δρ_max_ = 0.31 e Å^−^^3^
0 restraints	Δρ_min_ = −0.34 e Å^−^^3^

### Special details

**Table:**

Geometry. All e.s.d.'s (except the e.s.d. in the dihedral angle between two l.s. planes) are estimated using the full covariance matrix. The cell e.s.d.'s are taken into account individually in the estimation of e.s.d.'s in distances, angles and torsion angles; correlations between e.s.d.'s in cell parameters are only used when they are defined by crystal symmetry. An approximate (isotropic) treatment of cell e.s.d.'s is used for estimating e.s.d.'s involving l.s. planes.
Refinement. Refinement of *F*^2^ against ALL reflections. The weighted *R*-factor *wR* and goodness of fit *S* are based on *F*^2^, conventional *R*-factors *R* are based on *F*, with *F* set to zero for negative *F*^2^. The threshold expression of *F*^2^ > σ(*F*^2^) is used only for calculating *R*-factors(gt) *etc*. and is not relevant to the choice of reflections for refinement. *R*-factors based on *F*^2^ are statistically about twice as large as those based on *F*, and *R*- factors based on ALL data will be even larger.

### Fractional atomic coordinates and isotropic or equivalent isotropic displacement parameters (Å^2^)

**Table:**

	*x*	*y*	*z*	*U*_iso_*/*U*_eq_	
P1	0.19581 (4)	0.01563 (4)	0.03088 (2)	0.02376 (9)	
P1'	0.70287 (4)	0.52900 (4)	0.47655 (2)	0.02586 (9)	
O1	0.04093 (11)	0.08523 (11)	0.05977 (6)	0.0281 (2)	
O1'	0.55749 (12)	0.60327 (12)	0.44825 (6)	0.0307 (2)	
N1	0.23929 (14)	−0.13554 (13)	−0.01151 (7)	0.0257 (2)	
H1N	0.161 (2)	−0.138 (2)	−0.0202 (12)	0.044 (6)*	
N1'	0.72932 (14)	0.37818 (13)	0.52085 (7)	0.0273 (3)	
H1'N	0.649 (2)	0.371 (2)	0.5267 (11)	0.035 (5)*	
C1'	0.85909 (16)	0.27308 (15)	0.55182 (8)	0.0250 (3)	
C1	0.37739 (16)	−0.23456 (15)	−0.04656 (8)	0.0252 (3)	
O2	0.31273 (11)	−0.01688 (11)	0.08885 (6)	0.0275 (2)	
O2'	0.74025 (12)	0.62773 (11)	0.52562 (6)	0.0295 (2)	
C2	0.49794 (17)	−0.20592 (16)	−0.05467 (9)	0.0295 (3)	
H2A	0.4883	−0.1171	−0.0368	0.035*	
C2'	0.97362 (17)	0.30462 (16)	0.56286 (9)	0.0289 (3)	
H2'A	0.9657	0.3979	0.5494	0.035*	
O3	0.24525 (11)	0.11823 (11)	−0.02046 (6)	0.0272 (2)	
O3'	0.83944 (11)	0.49332 (11)	0.41738 (6)	0.0288 (2)	
C3'	1.09885 (17)	0.20008 (17)	0.59340 (9)	0.0306 (3)	
H3'A	1.1765	0.2220	0.6006	0.037*	
C3	0.63124 (18)	−0.30587 (18)	−0.08845 (9)	0.0339 (3)	
H3A	0.7126	−0.2853	−0.0938	0.041*	
C4'	1.11138 (18)	0.06399 (17)	0.61338 (9)	0.0316 (3)	
H4'A	1.1968	−0.0073	0.6347	0.038*	
C4	0.64678 (18)	−0.43601 (18)	−0.11448 (10)	0.0356 (3)	
H4A	0.7383	−0.5049	−0.1377	0.043*	
C5'	0.99799 (18)	0.03281 (17)	0.60188 (9)	0.0308 (3)	
H5'A	1.0073	−0.0610	0.6153	0.037*	
C5	0.52688 (18)	−0.46445 (17)	−0.10627 (9)	0.0333 (3)	
H5A	0.5381	−0.5541	−0.1239	0.040*	
C6'	0.87077 (16)	0.13484 (16)	0.57128 (8)	0.0262 (3)	
C6	0.39121 (17)	−0.36614 (16)	−0.07314 (8)	0.0277 (3)	
C7'	0.75118 (18)	0.09580 (17)	0.55958 (10)	0.0328 (3)	
H7'A	0.7800	−0.0047	0.5765	0.049*	
H7'B	0.7353	0.1048	0.5064	0.049*	
H7'C	0.6601	0.1618	0.5883	0.049*	
C7	0.26449 (18)	−0.40159 (17)	−0.06492 (10)	0.0341 (3)	
H7A	0.2955	−0.4972	−0.0866	0.051*	
H7B	0.1829	−0.3273	−0.0910	0.051*	
H7C	0.2325	−0.4034	−0.0118	0.051*	
C8	0.34238 (16)	−0.11692 (15)	0.14859 (8)	0.0252 (3)	
C8'	0.64352 (16)	0.71344 (16)	0.58442 (8)	0.0278 (3)	
C9	0.48097 (17)	−0.16872 (18)	0.17359 (9)	0.0326 (3)	
H9A	0.5504	−0.1406	0.1492	0.039*	
C9'	0.6185 (2)	0.6498 (2)	0.65008 (10)	0.0398 (4)	
H9'A	0.6587	0.5469	0.6547	0.048*	
C10	0.51614 (19)	−0.26239 (18)	0.23491 (10)	0.0373 (4)	
H10A	0.6106	−0.2987	0.2527	0.045*	
C10'	0.5339 (2)	0.7386 (3)	0.70923 (11)	0.0525 (5)	
H10B	0.5147	0.6966	0.7547	0.063*	
C11	0.41501 (19)	−0.30358 (17)	0.27055 (9)	0.0340 (3)	
H11A	0.4395	−0.3669	0.3129	0.041*	
C11'	0.4774 (2)	0.8877 (3)	0.70230 (11)	0.0527 (5)	
H11B	0.4206	0.9479	0.7433	0.063*	
C12	0.27790 (18)	−0.25192 (17)	0.24404 (9)	0.0313 (3)	
H12A	0.2087	−0.2807	0.2682	0.038*	
C12'	0.5030 (2)	0.9502 (2)	0.63599 (12)	0.0464 (5)	
H12B	0.4633	1.0531	0.6314	0.056*	
C13	0.24039 (16)	−0.15821 (16)	0.18236 (8)	0.0277 (3)	
H13A	0.1467	−0.1234	0.1639	0.033*	
C13'	0.58688 (17)	0.86229 (17)	0.57605 (10)	0.0324 (3)	
H13B	0.6048	0.9040	0.5302	0.039*	
C14'	0.88130 (16)	0.39496 (15)	0.35856 (8)	0.0272 (3)	
C14	0.16958 (16)	0.19573 (16)	−0.08173 (8)	0.0281 (3)	
C15	0.15880 (19)	0.12386 (19)	−0.14134 (9)	0.0349 (3)	
H15A	0.1947	0.0212	−0.1398	0.042*	
C15'	1.02970 (17)	0.32833 (17)	0.33465 (9)	0.0329 (3)	
H15B	1.0973	0.3447	0.3598	0.039*	
C16'	1.07788 (19)	0.23765 (19)	0.27364 (10)	0.0392 (4)	
H16A	1.1793	0.1920	0.2566	0.047*	
C16	0.0943 (2)	0.2051 (2)	−0.20348 (10)	0.0436 (4)	
H16B	0.0849	0.1576	−0.2447	0.052*	
C17'	0.9801 (2)	0.21280 (18)	0.23727 (10)	0.0394 (4)	
H17A	1.0138	0.1511	0.1950	0.047*	
C17	0.0437 (2)	0.3538 (2)	−0.20598 (11)	0.0478 (5)	
H17B	0.0012	0.4082	−0.2491	0.057*	
C18'	0.8327 (2)	0.27838 (18)	0.26281 (10)	0.0383 (4)	
H18A	0.7656	0.2602	0.2382	0.046*	
C18	0.0550 (2)	0.4240 (2)	−0.14562 (12)	0.0475 (5)	
H18B	0.0197	0.5266	−0.1474	0.057*	
C19'	0.78125 (18)	0.37031 (17)	0.32386 (9)	0.0330 (3)	
H19A	0.6800	0.4151	0.3413	0.040*	
C19	0.11776 (18)	0.34487 (18)	−0.08233 (10)	0.0364 (4)	
H19B	0.1248	0.3925	−0.0405	0.044*	

### Atomic displacement parameters (Å^2^)

**Table:**

	*U*^11^	*U*^22^	*U*^33^	*U*^12^	*U*^13^	*U*^23^
P1	0.02526 (18)	0.02339 (17)	0.02499 (18)	−0.01273 (15)	−0.00391 (14)	−0.00136 (13)
P1'	0.02571 (19)	0.02501 (18)	0.0303 (2)	−0.01476 (15)	−0.00277 (14)	0.00181 (14)
O1	0.0261 (5)	0.0289 (5)	0.0288 (5)	−0.0119 (4)	−0.0020 (4)	−0.0045 (4)
O1'	0.0272 (5)	0.0304 (5)	0.0366 (6)	−0.0152 (4)	−0.0050 (4)	0.0060 (4)
N1	0.0243 (6)	0.0249 (6)	0.0312 (6)	−0.0135 (5)	−0.0037 (5)	−0.0041 (5)
N1'	0.0264 (6)	0.0258 (6)	0.0349 (7)	−0.0165 (5)	−0.0044 (5)	0.0032 (5)
C1'	0.0265 (7)	0.0258 (7)	0.0235 (7)	−0.0128 (6)	−0.0009 (5)	−0.0005 (5)
C1	0.0261 (7)	0.0234 (7)	0.0249 (7)	−0.0099 (6)	−0.0046 (5)	0.0004 (5)
O2	0.0302 (5)	0.0293 (5)	0.0279 (5)	−0.0173 (4)	−0.0068 (4)	0.0020 (4)
O2'	0.0295 (5)	0.0261 (5)	0.0368 (6)	−0.0164 (4)	−0.0002 (4)	−0.0032 (4)
C2	0.0282 (7)	0.0278 (7)	0.0342 (8)	−0.0137 (6)	−0.0038 (6)	−0.0037 (6)
C2'	0.0318 (8)	0.0283 (7)	0.0313 (7)	−0.0177 (6)	−0.0043 (6)	0.0020 (6)
O3	0.0304 (5)	0.0257 (5)	0.0291 (5)	−0.0155 (4)	−0.0059 (4)	0.0020 (4)
O3'	0.0288 (5)	0.0296 (5)	0.0326 (5)	−0.0174 (4)	−0.0012 (4)	−0.0008 (4)
C3'	0.0304 (8)	0.0355 (8)	0.0293 (7)	−0.0178 (7)	−0.0047 (6)	0.0013 (6)
C3	0.0288 (8)	0.0376 (8)	0.0367 (8)	−0.0159 (7)	−0.0041 (6)	−0.0044 (7)
C4'	0.0329 (8)	0.0305 (7)	0.0294 (8)	−0.0126 (6)	−0.0050 (6)	0.0024 (6)
C4	0.0294 (8)	0.0339 (8)	0.0366 (8)	−0.0080 (7)	−0.0035 (6)	−0.0070 (7)
C5'	0.0378 (8)	0.0262 (7)	0.0289 (7)	−0.0155 (6)	−0.0017 (6)	0.0024 (6)
C5	0.0367 (8)	0.0260 (7)	0.0341 (8)	−0.0108 (6)	−0.0058 (6)	−0.0045 (6)
C6'	0.0305 (7)	0.0263 (7)	0.0247 (7)	−0.0160 (6)	0.0019 (6)	−0.0029 (5)
C6	0.0330 (8)	0.0244 (7)	0.0279 (7)	−0.0142 (6)	−0.0077 (6)	0.0012 (5)
C7'	0.0356 (8)	0.0286 (7)	0.0403 (9)	−0.0199 (7)	−0.0019 (7)	−0.0014 (6)
C7	0.0385 (9)	0.0277 (7)	0.0416 (9)	−0.0191 (7)	−0.0054 (7)	−0.0035 (6)
C8	0.0272 (7)	0.0227 (6)	0.0241 (7)	−0.0094 (6)	−0.0041 (5)	−0.0029 (5)
C8'	0.0255 (7)	0.0312 (7)	0.0302 (7)	−0.0156 (6)	−0.0046 (6)	−0.0007 (6)
C9	0.0283 (8)	0.0369 (8)	0.0343 (8)	−0.0158 (7)	−0.0053 (6)	−0.0014 (6)
C9'	0.0398 (9)	0.0477 (10)	0.0369 (9)	−0.0247 (8)	−0.0066 (7)	0.0098 (7)
C10	0.0328 (8)	0.0378 (9)	0.0394 (9)	−0.0126 (7)	−0.0139 (7)	0.0009 (7)
C10'	0.0483 (11)	0.0884 (16)	0.0309 (9)	−0.0407 (11)	−0.0025 (8)	0.0039 (9)
C11	0.0390 (9)	0.0298 (8)	0.0285 (8)	−0.0110 (7)	−0.0087 (6)	0.0019 (6)
C11'	0.0371 (10)	0.0828 (16)	0.0437 (11)	−0.0315 (10)	0.0076 (8)	−0.0273 (10)
C12	0.0336 (8)	0.0288 (7)	0.0294 (8)	−0.0129 (6)	−0.0008 (6)	−0.0008 (6)
C12'	0.0335 (9)	0.0430 (10)	0.0637 (12)	−0.0175 (8)	0.0009 (8)	−0.0190 (9)
C13	0.0243 (7)	0.0279 (7)	0.0289 (7)	−0.0101 (6)	−0.0028 (6)	−0.0010 (6)
C13'	0.0295 (8)	0.0309 (8)	0.0395 (9)	−0.0160 (6)	−0.0042 (6)	0.0004 (6)
C14'	0.0297 (7)	0.0232 (7)	0.0275 (7)	−0.0115 (6)	−0.0031 (6)	0.0034 (5)
C14	0.0252 (7)	0.0305 (7)	0.0294 (7)	−0.0141 (6)	−0.0017 (6)	0.0036 (6)
C15	0.0378 (9)	0.0385 (8)	0.0281 (8)	−0.0175 (7)	−0.0025 (6)	−0.0001 (6)
C15'	0.0280 (8)	0.0335 (8)	0.0365 (8)	−0.0143 (6)	−0.0028 (6)	0.0046 (6)
C16'	0.0334 (8)	0.0348 (8)	0.0404 (9)	−0.0096 (7)	0.0033 (7)	0.0022 (7)
C16	0.0443 (10)	0.0618 (12)	0.0293 (8)	−0.0286 (9)	−0.0056 (7)	0.0070 (8)
C17'	0.0479 (10)	0.0297 (8)	0.0316 (8)	−0.0108 (7)	−0.0016 (7)	0.0001 (6)
C17	0.0385 (10)	0.0624 (12)	0.0470 (11)	−0.0282 (9)	−0.0136 (8)	0.0266 (9)
C18'	0.0424 (9)	0.0358 (8)	0.0364 (9)	−0.0163 (7)	−0.0102 (7)	−0.0008 (7)
C18	0.0407 (10)	0.0379 (9)	0.0665 (13)	−0.0209 (8)	−0.0141 (9)	0.0212 (9)
C19'	0.0279 (8)	0.0326 (8)	0.0366 (8)	−0.0117 (6)	−0.0063 (6)	0.0004 (6)
C19	0.0340 (8)	0.0306 (8)	0.0480 (10)	−0.0179 (7)	−0.0058 (7)	0.0056 (7)

### Geometric parameters (Å, °)

**Table:**

P1—O1	1.4633 (11)	C8—C13	1.381 (2)
P1—O2	1.5799 (11)	C8—C9	1.387 (2)
P1—O3	1.5904 (11)	C8'—C13'	1.377 (2)
P1—N1	1.6284 (12)	C8'—C9'	1.378 (2)
P1'—O1'	1.4626 (11)	C9—C10	1.386 (2)
P1'—O3'	1.5818 (11)	C9—H9A	0.9500
P1'—O2'	1.5852 (11)	C9'—C10'	1.385 (3)
P1'—N1'	1.6290 (13)	C9'—H9'A	0.9500
N1—C1	1.4181 (19)	C10—C11	1.385 (2)
N1—H1N	0.85 (2)	C10—H10A	0.9500
N1'—C1'	1.4207 (19)	C10'—C11'	1.377 (3)
N1'—H1'N	0.86 (2)	C10'—H10B	0.9500
C1'—C2'	1.396 (2)	C11—C12	1.385 (2)
C1'—C6'	1.4060 (19)	C11—H11A	0.9500
C1—C2	1.396 (2)	C11'—C12'	1.384 (3)
C1—C6	1.4097 (19)	C11'—H11B	0.9500
O2—C8	1.4024 (17)	C12—C13	1.392 (2)
O2'—C8'	1.4041 (18)	C12—H12A	0.9500
C2—C3	1.382 (2)	C12'—C13'	1.390 (2)
C2—H2A	0.9500	C12'—H12B	0.9500
C2'—C3'	1.386 (2)	C13—H13A	0.9500
C2'—H2'A	0.9500	C13'—H13B	0.9500
O3—C14	1.4011 (18)	C14'—C19'	1.383 (2)
O3'—C14'	1.4028 (18)	C14'—C15'	1.385 (2)
C3'—C4'	1.383 (2)	C14—C19	1.381 (2)
C3'—H3'A	0.9500	C14—C15	1.382 (2)
C3—C4	1.385 (2)	C15—C16	1.388 (2)
C3—H3A	0.9500	C15—H15A	0.9500
C4'—C5'	1.385 (2)	C15'—C16'	1.382 (2)
C4'—H4'A	0.9500	C15'—H15B	0.9500
C4—C5	1.388 (2)	C16'—C17'	1.380 (3)
C4—H4A	0.9500	C16'—H16A	0.9500
C5'—C6'	1.392 (2)	C16—C17	1.378 (3)
C5'—H5'A	0.9500	C16—H16B	0.9500
C5—C6	1.387 (2)	C17'—C18'	1.384 (3)
C5—H5A	0.9500	C17'—H17A	0.9500
C6'—C7'	1.500 (2)	C17—C18	1.385 (3)
C6—C7	1.502 (2)	C17—H17B	0.9500
C7'—H7'A	0.9800	C18'—C19'	1.388 (2)
C7'—H7'B	0.9800	C18'—H18A	0.9500
C7'—H7'C	0.9800	C18—C19	1.393 (3)
C7—H7A	0.9800	C18—H18B	0.9500
C7—H7B	0.9800	C19'—H19A	0.9500
C7—H7C	0.9800	C19—H19B	0.9500
			
O1—P1—O2	116.87 (6)	C13—C8—O2	123.06 (13)
O1—P1—O3	114.56 (6)	C9—C8—O2	115.18 (13)
O2—P1—O3	93.94 (5)	C13'—C8'—C9'	121.93 (16)
O1—P1—N1	111.23 (6)	C13'—C8'—O2'	117.01 (14)
O2—P1—N1	108.09 (6)	C9'—C8'—O2'	120.77 (14)
O3—P1—N1	110.93 (6)	C10—C9—C8	118.70 (15)
O1'—P1'—O3'	116.62 (6)	C10—C9—H9A	120.7
O1'—P1'—O2'	114.45 (6)	C8—C9—H9A	120.7
O3'—P1'—O2'	93.95 (6)	C8'—C9'—C10'	118.82 (18)
O1'—P1'—N1'	111.98 (7)	C8'—C9'—H9'A	120.6
O3'—P1'—N1'	107.75 (6)	C10'—C9'—H9'A	120.6
O2'—P1'—N1'	110.71 (6)	C11—C10—C9	120.72 (15)
C1—N1—P1	128.61 (10)	C11—C10—H10A	119.6
C1—N1—H1N	120.6 (14)	C9—C10—H10A	119.6
P1—N1—H1N	109.1 (14)	C11'—C10'—C9'	120.18 (18)
C1'—N1'—P1'	128.14 (10)	C11'—C10'—H10B	119.9
C1'—N1'—H1'N	121.4 (13)	C9'—C10'—H10B	119.9
P1'—N1'—H1'N	110.3 (13)	C10—C11—C12	119.58 (15)
C2'—C1'—C6'	120.07 (13)	C10—C11—H11A	120.2
C2'—C1'—N1'	121.39 (13)	C12—C11—H11A	120.2
C6'—C1'—N1'	118.54 (13)	C10'—C11'—C12'	120.42 (18)
C2—C1—C6	119.91 (14)	C10'—C11'—H11B	119.8
C2—C1—N1	122.19 (13)	C12'—C11'—H11B	119.8
C6—C1—N1	117.90 (13)	C11—C12—C13	120.69 (15)
C8—O2—P1	125.79 (9)	C11—C12—H12A	119.7
C8'—O2'—P1'	122.76 (9)	C13—C12—H12A	119.7
C3—C2—C1	120.49 (14)	C11'—C12'—C13'	119.92 (18)
C3—C2—H2A	119.8	C11'—C12'—H12B	120.0
C1—C2—H2A	119.8	C13'—C12'—H12B	120.0
C3'—C2'—C1'	120.16 (14)	C8—C13—C12	118.57 (14)
C3'—C2'—H2'A	119.9	C8—C13—H13A	120.7
C1'—C2'—H2'A	119.9	C12—C13—H13A	120.7
C14—O3—P1	122.12 (9)	C8'—C13'—C12'	118.72 (17)
C14'—O3'—P1'	124.49 (9)	C8'—C13'—H13B	120.6
C4'—C3'—C2'	120.41 (14)	C12'—C13'—H13B	120.6
C4'—C3'—H3'A	119.8	C19'—C14'—C15'	121.58 (15)
C2'—C3'—H3'A	119.8	C19'—C14'—O3'	122.75 (14)
C2—C3—C4	120.28 (15)	C15'—C14'—O3'	115.60 (13)
C2—C3—H3A	119.9	C19—C14—C15	121.98 (15)
C4—C3—H3A	119.9	C19—C14—O3	116.85 (14)
C3'—C4'—C5'	119.25 (14)	C15—C14—O3	120.95 (14)
C3'—C4'—H4'A	120.4	C14—C15—C16	118.51 (16)
C5'—C4'—H4'A	120.4	C14—C15—H15A	120.7
C3—C4—C5	119.17 (15)	C16—C15—H15A	120.7
C3—C4—H4A	120.4	C16'—C15'—C14'	118.97 (15)
C5—C4—H4A	120.4	C16'—C15'—H15B	120.5
C4'—C5'—C6'	121.97 (14)	C14'—C15'—H15B	120.5
C4'—C5'—H5'A	119.0	C17'—C16'—C15'	120.66 (16)
C6'—C5'—H5'A	119.0	C17'—C16'—H16A	119.7
C6—C5—C4	122.08 (15)	C15'—C16'—H16A	119.7
C6—C5—H5A	119.0	C17—C16—C15	120.67 (18)
C4—C5—H5A	119.0	C17—C16—H16B	119.7
C5'—C6'—C1'	118.13 (13)	C15—C16—H16B	119.7
C5'—C6'—C7'	120.03 (13)	C16'—C17'—C18'	119.49 (16)
C1'—C6'—C7'	121.83 (14)	C16'—C17'—H17A	120.3
C5—C6—C1	118.07 (14)	C18'—C17'—H17A	120.3
C5—C6—C7	120.38 (14)	C16—C17—C18	120.00 (17)
C1—C6—C7	121.54 (14)	C16—C17—H17B	120.0
C6'—C7'—H7'A	109.5	C18—C17—H17B	120.0
C6'—C7'—H7'B	109.5	C17'—C18'—C19'	121.02 (16)
H7'A—C7'—H7'B	109.5	C17'—C18'—H18A	119.5
C6'—C7'—H7'C	109.5	C19'—C18'—H18A	119.5
H7'A—C7'—H7'C	109.5	C17—C18—C19	120.29 (17)
H7'B—C7'—H7'C	109.5	C17—C18—H18B	119.9
C6—C7—H7A	109.5	C19—C18—H18B	119.9
C6—C7—H7B	109.5	C14'—C19'—C18'	118.25 (15)
H7A—C7—H7B	109.5	C14'—C19'—H19A	120.9
C6—C7—H7C	109.5	C18'—C19'—H19A	120.9
H7A—C7—H7C	109.5	C14—C19—C18	118.53 (17)
H7B—C7—H7C	109.5	C14—C19—H19B	120.7
C13—C8—C9	121.73 (14)	C18—C19—H19B	120.7
			
O1—P1—N1—C1	−179.73 (12)	C2—C1—C6—C7	179.49 (14)
O2—P1—N1—C1	−50.16 (14)	N1—C1—C6—C7	0.0 (2)
O3—P1—N1—C1	51.51 (14)	P1—O2—C8—C13	−25.96 (19)
O1'—P1'—N1'—C1'	175.80 (12)	P1—O2—C8—C9	156.16 (11)
O3'—P1'—N1'—C1'	46.27 (14)	P1'—O2'—C8'—C13'	−114.99 (13)
O2'—P1'—N1'—C1'	−55.17 (14)	P1'—O2'—C8'—C9'	71.06 (17)
P1'—N1'—C1'—C2'	15.3 (2)	C13—C8—C9—C10	−1.1 (2)
P1'—N1'—C1'—C6'	−164.52 (11)	O2—C8—C9—C10	176.79 (13)
P1—N1—C1—C2	−5.7 (2)	C13'—C8'—C9'—C10'	0.0 (3)
P1—N1—C1—C6	173.76 (11)	O2'—C8'—C9'—C10'	173.69 (15)
O1—P1—O2—C8	66.73 (13)	C8—C9—C10—C11	0.0 (2)
O3—P1—O2—C8	−173.14 (11)	C8'—C9'—C10'—C11'	−0.7 (3)
N1—P1—O2—C8	−59.61 (12)	C9—C10—C11—C12	0.8 (2)
O1'—P1'—O2'—C8'	48.83 (13)	C9'—C10'—C11'—C12'	0.9 (3)
O3'—P1'—O2'—C8'	170.49 (11)	C10—C11—C12—C13	−0.5 (2)
N1'—P1'—O2'—C8'	−78.86 (12)	C10'—C11'—C12'—C13'	−0.4 (3)
C6—C1—C2—C3	−0.2 (2)	C9—C8—C13—C12	1.4 (2)
N1—C1—C2—C3	179.30 (14)	O2—C8—C13—C12	−176.37 (13)
C6'—C1'—C2'—C3'	−0.3 (2)	C11—C12—C13—C8	−0.5 (2)
N1'—C1'—C2'—C3'	179.81 (14)	C9'—C8'—C13'—C12'	0.4 (2)
O1—P1—O3—C14	−52.23 (12)	O2'—C8'—C13'—C12'	−173.45 (14)
O2—P1—O3—C14	−174.20 (11)	C11'—C12'—C13'—C8'	−0.2 (3)
N1—P1—O3—C14	74.72 (12)	P1'—O3'—C14'—C19'	31.11 (19)
O1'—P1'—O3'—C14'	−66.01 (12)	P1'—O3'—C14'—C15'	−151.86 (11)
O2'—P1'—O3'—C14'	174.08 (11)	P1—O3—C14—C19	123.66 (13)
N1'—P1'—O3'—C14'	60.86 (12)	P1—O3—C14—C15	−61.57 (18)
C1'—C2'—C3'—C4'	−0.2 (2)	C19—C14—C15—C16	0.3 (2)
C1—C2—C3—C4	−0.2 (2)	O3—C14—C15—C16	−174.23 (14)
C2'—C3'—C4'—C5'	0.6 (2)	C19'—C14'—C15'—C16'	1.4 (2)
C2—C3—C4—C5	0.1 (3)	O3'—C14'—C15'—C16'	−175.64 (14)
C3'—C4'—C5'—C6'	−0.5 (2)	C14'—C15'—C16'—C17'	−0.5 (2)
C3—C4—C5—C6	0.4 (3)	C14—C15—C16—C17	0.8 (3)
C4'—C5'—C6'—C1'	0.0 (2)	C15'—C16'—C17'—C18'	−0.7 (3)
C4'—C5'—C6'—C7'	179.74 (15)	C15—C16—C17—C18	−1.0 (3)
C2'—C1'—C6'—C5'	0.4 (2)	C16'—C17'—C18'—C19'	0.9 (3)
N1'—C1'—C6'—C5'	−179.72 (13)	C16—C17—C18—C19	0.3 (3)
C2'—C1'—C6'—C7'	−179.31 (14)	C15'—C14'—C19'—C18'	−1.2 (2)
N1'—C1'—C6'—C7'	0.5 (2)	O3'—C14'—C19'—C18'	175.61 (14)
C4—C5—C6—C1	−0.7 (2)	C17'—C18'—C19'—C14'	0.1 (2)
C4—C5—C6—C7	−179.63 (15)	C15—C14—C19—C18	−1.0 (2)
C2—C1—C6—C5	0.6 (2)	O3—C14—C19—C18	173.70 (15)
N1—C1—C6—C5	−178.87 (13)	C17—C18—C19—C14	0.7 (3)

### Hydrogen-bond geometry (Å, °)

**Table:**

*D*—H···*A*	*D*—H	H···*A*	*D*···*A*	*D*—H···*A*
N1—H1N···O1^i^	0.85 (2)	2.09 (2)	2.903 (1)	162 (1)
N1'—H1'N···O1'^ii^	0.86 (2)	2.02 (2)	2.867 (1)	167 (1)

Symmetry codes: (i) −*x*, −*y*, −*z*; (ii) −*x*+1, −*y*+1, −*z*+1.
